# Genome‐based prediction of Bayesian linear and non‐linear regression models for ordinal data

**DOI:** 10.1002/tpg2.20021

**Published:** 2020-05-14

**Authors:** Paulino Pérez‐Rodríguez, Samuel Flores‐Galarza, Humberto Vaquera‐Huerta, David Hebert del Valle‐Paniagua, Osval A. Montesinos‐López, José Crossa

**Affiliations:** ^1^ Colegio de Postgraduados CP 56230, Montecillos, Edo. de México; ^2^ Biometrics and Statistics Unit International Maize and Wheat Improvement Center (CIMMYT) Apdo. Postal 6–641, 06600, Cd. de México; ^3^ Facultad de Telemática, Universidad de Colima Colima 28040 México

## Abstract

Linear and non‐linear models used in applications of genomic selection (GS) can fit different types of responses (e.g., continuous, ordinal, binary). In recent years, several genomic‐enabled prediction models have been developed for predicting complex traits in genomic‐assisted animal and plant breeding. These models include linear, non‐linear and non‐parametric models, mostly for continuous responses and less frequently for categorical responses. Several linear and non‐linear models are special cases of a more general family of statistical models known as artificial neural networks, which provide better prediction ability than other models. In this paper, we propose a Bayesian Regularized Neural Network (BRNNO) for modelling ordinal data. The proposed model was fitted using a Bayesian framework; we used the data augmentation algorithm to facilitate computations. The proposed model was fitted using the Gibbs Maximum a Posteriori and Generalized EM algorithm implemented by combining code written in C and R programming languages. The new model was tested with two real maize datasets evaluated for Septoria and GLS diseases and was compared with the Bayesian Ordered Probit Model (BOPM). Results indicated that the BRNNO model performed better in terms of genomic‐based prediction than the BOPM model.

**Abbreviations**
BGLR: Bayesian Generalized Linear Regression
BOPM: Bayesian ordered probit model
BRNNO: Bayesian Regularized Neural Network for Ordinal data
BS: Brier Score
GBS: Genotype by Sequencing
GEM: Generalized EM algorithm
GLS: Gray Leaf Spot
GMAP: Gibbs Maximum A Posteriori
GS: Genomic Selection
MAE: Mean Absolute Error
MCMC: Markov Chain Monte Carlo
MER: Misclassification Error Rate
RMSE: Root Mean Square Error
SHL: Single Hidden Layer
SHLNN: Single Hidden Layer Neural Network

## 1 INTRODUCTION

In genomic selection (GS) and genomic prediction (GP), all markers are fitted simultaneously to the training and testing populations. Practical and simulation studies have favored the use of GS as a way to increase genetic gains in less time (Crossa et al., 2017). For continuous traits, models have been developed to regress phenotypes on all markers using a linear model (Meuwissen, Hayes, & Goddard, 2001). However, in plant and animal breeding, the response variables in many traits are discrete counts (*y *= 0, 1, 2, …). In classical probability theory, counts could indicate a binomial, multinomial or a Poisson distribution. For smaller counts, data analysts recommend using logarithmic or square root transformations. In GS it is still common practice to apply linear regression models to categorical data or transformed data (Montesinos‐López et al., 2015a). Transformations do not consider many distributions (of count data) as positively skewed: several observations have a value of 0, and a high number of 0 values in the data does not allow a skewed distribution to be transformed into a normal distribution; it is also possible that the regression model will produce negative predicted values. When a transformation is used, it is not always possible to have normally distributed transformed data, and often times, transformations are counterproductive (Stroup, 2012).

Ordinal data are very common in many research fields, such as econometrics, finance, agriculture, animal and plant breeding, genetics, etc. In agricultural applications, resistance/susceptibility to diseases is usually measured on an ordinal scale (e.g., 1 = no disease, 2 = low infection, 3 = moderate infection, 4 = high infection, and 5 = totally infected). With data on this scale, it is possible to obtain relative or absolute frequencies, but the usual sample mean or sample standard deviation cannot be obtained, since the distance between classes is not defined (Stevens, 1946). An important application with ordinal data is ordinal regression, where a response variable that is measured on an ordinal scale is predicted by using several covariates. Ordinal regression has been widely used in plant and animal breeding (e.g., Gianola, 1982) and is mainly based on linear mixed models. However, ignoring the ordinal nature of the data can cause several problems related to biased estimates, which could potentially lead to wrong and misleading conclusions (Montesinos‐López et al., 2015a). In plant breeding, several economically important traits are categorical, and in general, threshold models have not been considered in GS. One of the first studies that introduced the threshold model to GS incorporating genomic × environment interaction was Montesinos‐López et al. (2015a) when 278 maize lines scored for resistance to gray leaf spot (GLS) were measured using an ordered categorical scale in three environments.

In GS, a response variable is predicted based on dense molecular markers. Most of the models used to predict the response variable are linear, and the following assumptions hold: (1) the response variable is normally distributed, (2) the variance of the residuals is constant, and (3) the expected value of the response variable is a linear function of covariates (Montesinos‐López et al., 2015a; Pérez‐Rodríguez et al., 2012). Recently, linear models used in GS have been extended to model ordered phenotypical categories (e.g., Montesinos‐López et al., 2015a; Montesinos‐López, Montesinos‐López, Crossa, Burgueño, & Eskridge, 2015b; Wang et al., 2013).

In order to make predictions on new data, machine learning builds models from sample data using several algorithms (Samuel, 1959). There are some applications of machine learning in genomic selection (González‐Camacho et al., 2012; González‐Camacho et al., 2018) with connected network architecture, which is a type of machine learning algorithm that uses an artificial neural network with input, hidden and output layers linked non‐linearly. The layers consist of stages of non‐linear data transformations. Non‐linear kernel‐based models and neural networks have also been used in the context of GS for continuous traits with promising results (González‐Camacho et al., 2012, 2018; Pérez‐Rodríguez et al., 2012). Recently, Montesinos‐López et al. (2019) compared genome‐based prediction accuracies for ordinal traits using several deep machine learning methods and the threshold model for ordinal data on an extensive number of ordinal data; the authors found that the threshold genomic best linear unbiased prediction was the most consistent model in terms of genomic‐enabled prediction accuracy. However, the scientific literature does not show results on many non‐linear GS models and methods for categorical data.

Based on the previous considerations, and motivated by the fact that (1) artificial neural networks are considered universal approximations, which in some cases exhibit better predictive ability than linear and non‐linear models, and that (2) several traits commonly used in GS are measured on an ordinal scale, the main objective of this study was to introduce a Bayesian Regularized Neural Network for Ordinal data (BRNNO) with a Single Hidden Layer (SHL). We used a data augmentation algorithm (Tanner, 1993) to make part of the computations feasible. We assumed that the process that leads to the observed response variable is a latent variable (unobserved), which is a continuous random variable that follows a standard normal distribution (e.g., Albert & Chib, 1993; Gianola, 1982; Montesinos‐López et al., 2015a). We combined Markov Chain Monte Carlo (MCMC) simulation and EM with other optimization algorithms that allowed us to fit the proposed model. The BRNNO model can also be considered a generalization of the probit ordered regression in the context of non‐linear models and can also be extended to generalize the logit ordered regression model (Montesinos‐López et al., 2015b).

**Core Ideas**

- Neural networks are universal approximation machines that can be adapted to predict ordinal responses
- Neural networks outperform the predictive power of linear models
- Data augmentation is a valuable tool for fitting neural network models

This paper is organized as follows: In the Materials and Methods section we introduce the Bayesian ordered probit model (BOPM) and the Bayesian ordered logit model (BOLM) for ordinal data; next, we introduce the Bayesian Regularized Neural Network for Ordinal data (BRNNO) and describe the procedures used to assess the predictive power of the proposed model. Then, we present an application of the BRNNO using two real datasets for wheat diseases from CIMMYT maize and wheat breeding trials (https://www.cimmyt.org). Finally, we present the results and discussion.

## 2 MATERIALS AND METHODS

### 2.1 Statistical models

According to Albert and Chib (1993), suppose that $Y_{1},\ldots ,Y_{n}$ are observed and $Y_{i}$ can take observations on *K* ordered values, that is, $Y_{i}\in \left\{1,\ldots ,K\right\}$, 1<2<···<K. Furthermore, suppose that we are interested in modelling the probabilities, $p_{ij}=\;P\left(\;Y_{i}=\;j\right)$, j=1,…,K. Probabilities $p_{ij}$ can be modelled using covariates $x_{i}^{t}=\left(x_{i1}\;,\ldots ,x_{ip}\right)$ with regression techniques, which leads to several very well‐known statistical models, e.g., the probit model or the ordered logit model, among others. Below we briefly discuss these two models from a Bayesian perspective.

### 2.2 Linear models: Bayesian ordered probit model, Bayesian ordered logistic model

#### 2.2.1 Bayesian ordered probit model (BOPM)

Albert and Chib (1993) assume that there is a unobserved random variable $Z_{i}$ normally distributed with mean $x_{i}^{t}\beta$ and variance 1, where $x_{i}^{t}=\left(x_{i1},\ldots ,x_{ip}\right)\;,$ a *p*‐dimensional vector of input covariates andβis a vector of unknown regression coefficients of dimensions p×1. We observe that $Y_{i}=\;j$ if $\lambda_{j-1}<Z_{i}<\lambda_{j}$ with $\lambda_{0}=\;-\infty ,\;\lambda_{K}=\;\infty$, $\lambda_{1},\ldots ,\lambda_{K-1\;}$ are a set of unknown thresholds; note also that $\lambda_{0}\leq \lambda_{1}\leq \cdots \leq \lambda_{K}$. The latent variable $Z_{i},$ also known as liability (Gianola, 1982), can be represented using the regression framework, that is:

(1) $$\;Z_{i}=x_{i}^{t}\;\beta +e_{i}$$

where $e_{i}$ is a random normal variable with mean 0 and variance 1. Using this representation, it can be shown that:

(2) $$P\left(\;Y_{i}=\;j\right)=\;\;\Phi \left(x_{i}^{t}\beta -\lambda_{j}\right)-\Phi \left(x_{i}^{t}\beta -\lambda_{j-1}\right)$$

(3) $$P\left(Y_{i}\leq j\right)\;=\;\Phi \left(\lambda_{j}-x_{i}^{t}\beta \right)$$

with Φ(·) as the standard normal cumulative distribution. Albert and Chib (1993) assigned a diffuse prior for (β,λ) and showed that all conditional distributions necessary to implement the Gibbs Sampler algorithm (Geman & Geman, 1984) have closed form and can be used to draw samples from the posterior distribution p(β,λ|data).

#### 2.2.2 Bayesian ordered logit model (BOLM)

Montesinos‐López et al. (2015b) argue that the ordinal logistic regression model is often preferred over the ordinal probit model because it provides regression coefficients that can be more easily interpreted as the regression coefficients are connected with the odds ratio. Montesinos‐López et al. (2015b) proposed using a latent variable (liability) with logistic distribution, which can be represented as follows:

(4) $$\;Z_{i}=x_{i}^{t}\;\beta +{\tilde{e}}_{i}$$

where ${\tilde{e}}_{i}$ is the standard logistic random variable; therefore, the probabilities $P(\;Y_{i}=\;j)$ are computed based on the distribution function of a logistic random variable. The authors propose using Póyla‐Gamma data augmentation, which facilitates the computations when sampling from the posterior distribution of the parameters of interest with the Gibbs sampler (Geman & Geman, 1984).

### 2.3 The proposed non‐linear model: Bayesian Regularized Neural Networks for Ordinal data (BRNNO)

**FIGURE 1:**
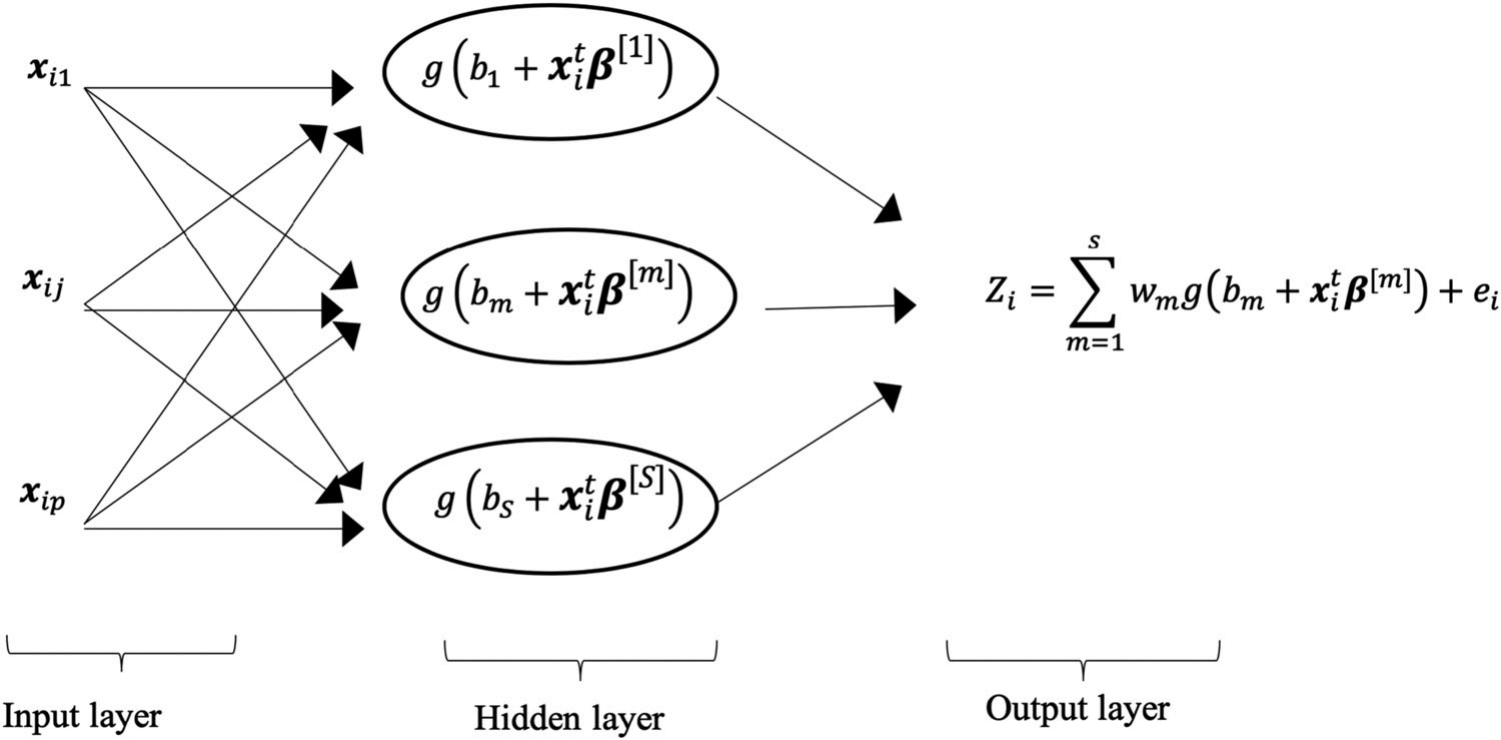
Structure of a single‐layer feed forward neural network (SLNN) adapted from González‐Camacho et al. (2012) and Pérez‐Rodríguez et al. (2013) for ordinal data using the latent variable approach

Following Albert and Chib (1993), Gianola (1982), González‐Camacho et al. (2012) and Pérez‐Rodríguez, Gianola, Weigel, Rosa, and Crossa (2013), in this study, we propose using the latent variable approach in order to model ordinal data with a Single Hidden Layer Neural Network (SHLNN), as shown in Figure 1. The structure of the neural network was analogous to that shown in Pérez‐Rodríguez et al. (2013), that is, the input layer has the input variables $x_{i}^{t}=\left(x_{i1},\ldots ,x_{ip}\;\right)$, which were then combined with linear inputs in neuron m=1,…,S and transformed by applying a non‐linear activation function g(·) in the hidden layer and then combined linearly in the output layer to regress the latent variable, that is:

(5) $$\;Z_{i}=\sum_{m=1}^{s}w_{m}\;g\left(b_{m}+x_{i}^{t}\beta^{\left[m\right]}\right)+e_{i}$$

The activation function is a function that maps the inputs from the real line into the bounded open interval (−1,1), for example, the tangent hyperbolic function tanh(·) is given by $g\;\left(u\right)=\frac{2}{1+\exp(-2u)}\;-1.\;$There are several options for activation functions, but tanh(·) is a popular alternative implemented in most software packages that fits neural networks. The output from equation (5) is related to the observed data using the approach employed in the probit and logit ordered model, that is, $Y_{i}=\;j$ if $\lambda_{j-1}<Z_{i}<\lambda_{j}$ with $\lambda_{0}=\;-\infty ,\;\lambda_{K}=\;\infty$, $\lambda_{1},\ldots ,\lambda_{K-1\;}$ the set of unknown thresholds. By using this approach, the probabilities given in equations (2) and (3) can be computed as follows:

(6) $$\begin{array}{ccc} P\left(\;Y_{i}=\;j\right)\; & = & \;\Phi \left(\sum_{m=1}^{s}w_{m}g\left(b_{m}+x_{i}^{t}\beta^{\left[m\right]}\right)-\lambda_{j}\right)\\ & & -\;\Phi \left(\sum_{m=1}^{s}w_{m}g\left(b_{m}+x_{i}^{t}\beta^{\left[m\right]}\right)-\lambda_{j-1}\right) \end{array}$$

(7) $$P\left(Y_{i}\leq j\right)\;=\;\Phi \left(\lambda_{j}-\sum_{m=1}^{s}w_{m}g\left(b_{m}+x_{i}^{t}\beta^{\left[m\right]}\right)\right)$$

Note that the ordered probit model (Albert & Chib, 1993) is a special case of the single layer perceptron, which also is a special case of the proposed Single Layer Neural Network, which is obtained by setting the number of neurons equal to 1 (S=1), $w_{1}=\;1$, g(u)=u (identity function), $b_{1}=\;0$, $\beta \;=\beta^{\left[1\right]}\;$. A Single Hidden Layer Neural Network that generalizes the ordered logit model proposed by Montesinos‐López et al. (2015b) can be obtained replacing the random error term in equation (5) by a random error term with standard logistic distribution.

The *BRNNO* model can be fitted using the Generalized Expectation‐Maximization algorithm (Kärkkäinen & Sillanpää, 2013; Neal & Hinton, 1998) or by using Bayesian methods. Let $\theta =(w_{1},\;\ldots ,w_{S};b_{1},\ldots ,b_{S};\beta^{\left[1\right]},\ldots ,\beta^{\left[S\right]})$, the vector of weights, biases and connection strengths, and let $p(\theta |\sigma_{\;\theta }^{2})$ be the prior distribution assigned to the elements of this vector. Here we assume that $p\;\left(\theta |\sigma_{\theta }^{2}\right)=\;MN\left(0,\sigma_{\theta }^{2}I\right)$, where MN(μ,Σ) stands for multivariate normal distribution with mean ***μ*** and variance‐covariance matrix Σ; in this case, μ=0 and $\Sigma \;=\sigma_{\theta }^{2}\;I$, with $\sigma_{\theta }^{2}$ a variance parameter common to all the elements in ***θ***, assuming for the moment that $\sigma_{\theta }^{2}$ is known. In the data augmentation framework for ordinal data (e.g., Albert & Chib, 1993), the joint posterior distribution of $\left\{\theta ,\lambda_{1},\ldots ,\lambda_{K-1},Z_{1},\ldots ,Z_{n}\right\}$, given the observed data, can be obtained as follows:

$$\;\begin{array}{ccc} p\left(\theta ,\lambda ,Z|y\right) & \propto & p\left(y|Z,\lambda ,\theta \right)p\left(Z|\lambda ,\theta \right)p\left(\theta ,\lambda |\sigma_{\theta }^{2}\right)\\ & = & p\left(y|Z,\lambda \right)p\left(Z|\theta \right)p\left(\theta |\sigma_{\theta }^{2}\right)p\left(\lambda \right) \end{array}\;$$

By assigning a diffuse prior for p(λ), this leads to:

(8) $$\begin{array}{ccc} p\left(\theta ,\lambda ,Z|y\right) & \propto & \prod_{i\;=\;1}^{n}\left[\varphi \left(z_{i};\sum_{m\;=\;1}^{s}w_{m}g\left(b_{m}+x_{i}^{t}\beta^{\left[m\right]}\right),1\right)\right.\\ & & \;\left.\times \sum_{j\;=\;1}^{K}1\left(\;Y_{i}=\;j\right)1\left(\lambda_{j-1}<Z_{i}\leq \lambda_{j}\right)\right]\\ & & \;\times p\left(\theta |\sigma_{\theta }^{2}\right)p\left(\lambda \right) \end{array}$$

where $\varphi (;\mu ,\sigma^{2})$ is the density function of a normal random variable with mean μ and variance σ^2^, and 1(X∈A) is the indicator function that takes a value of 1 if *X* is contained in the *A* set, and a value of 0 otherwise. Note that the joint posterior distribution of the parameters of interest does not have a closed form, so we must use numerical algorithms and simulation techniques to fit this model. In order to ensure the identification of the ***λ*** parameters, λ_1_ is set to 0 (Albert & Chib, 1993).

Next, we describe two numerical algorithms that can be employed to fit the BRNNO model: GMAP and GEM (Generalized EM algorithm). The GMAP algorithm (Kim, Hall, & Li, 2009) combines the Gibbs Sampler algorithm (G = Gibbs; see Geman & Geman, 1984) and an optimization algorithm to obtain the posterior modes of some parameters of interest in the model (MAP = Maximum A Posteriori). Pursuant to Kim et al. (2009), it is necessary to divide the parameters into two parts, which are labeled as ‘tractable’ and ‘intractable’. Tractable parameters are those whose full conditional distributions have a closed form, whereas the remaining parameters are ‘intractable’. Thus, ‘tractable’ parameters can be sampled by using the Gibbs sampler and ‘intractable’ parameters are estimated by MAP and G; MAP steps are repeated until convergence. As for the problem of interest, the tractable parameters are the latent variables and the thresholds {Z,λ}, while the intractable parameters are the weights, biases, connection strengths and variance parameter $\sigma_{\theta }^{2}$, that is $\left\{\theta ,\sigma_{\theta }^{2}\right\}$.

Next, we derive the full conditional distributions for tractable parameters and briefly explain how to obtain the posterior modes of intractable parameters. Appendix 1 shows computational details for implementing the GMAP algorithm.

- ***Conditional distributions of ‘tractable’ parameters***

a.1. *Conditional distribution of latent variables*, $p(Z_{i}|\;Y_{i}=\;j,\mathrm{else}),\;i\;=\;1,\ldots ,n$.

The fully conditional distribution of the latent variables can be obtained from (8); it has closed forms and can be obtained as follows:

(9) $$\begin{array}{ccc} p\left(Z_{i}|\;Y_{i}=\;j,\;else\right) & \propto & \varphi \left(Z_{i};\sum_{m\;=\;1}^{s}w_{m}g\left(b_{m}+x_{i}^{t}\beta^{\left[m\right]}\right),1\right)\\ & & \;\times \sum_{j\;=\;1}^{K}1\left(\;Y_{i}=\;j\right)1\left(\lambda_{j-1}<Z_{i}\leq \lambda_{j}\right), \end{array}$$

which corresponds to a truncated normal random variable truncated at the left (right) by $\lambda_{j-1}$ ($\lambda_{j})$, location parameter $\sum_{m\;=\;1}^{s}w_{m}g\left(b_{m}+x_{i}^{t}\beta^{\left[m\right]}\right)$ and scale parameter 1.

*a.2. Conditional distribution of thresholds*, p(λ|else)

The fully conditional distribution of $\lambda_{j}|else$ is proportional to:

(10) $$\begin{array}{ccc} p\left(\lambda_{j}|else\right) & \propto & \prod_{i\;=\;1}^{n}\left[1\left(\;Y_{i}=\;j\right)1\left(\lambda_{j-1}<Z_{i}<\lambda_{j}\right)\right.\\ & & +\;\left.1\left(\;Y_{i}=\;j+1\right)1\left(\lambda_{j}<Z_{i}<\lambda_{j+1}\right)\right],\\ & & \;\;j\;=\;2,..,K, \end{array}$$

which corresponds to a uniform distribution in the [a,b] interval, where $a\;=\max\{\max\left\{Z_{i}:Y_{i}=j\right\},\lambda_{j-1\;}\}\;$, $b\;=\min\{\min\left\{Z_{i}:Y_{i}=j+1\right\},\lambda_{j+1\;}\}\;$; see Albert and Chib (1993) for more details.

- ***Conditional posterior modes of ‘intractable’ parameters***

The set of intractable parameters is $\left\{\theta ,\sigma_{\theta }^{2}\right\}$. From equation (5), and by taking into account the prior distribution assigned to ***θ***, it is clear that the problem reduces to fitting a Bayesian Regularized Neural Network with a Single Hidden Layer; the details of the algorithm can be found elsewhere, such as in Foresee and Hagan (1997), Gianola, Okut, Weigel, and Rosa (2011), Okut, Gianola, Rosa, and Weigel (2011), and Pérez‐Rodríguez et al. (2013), among others. The algorithms used to fit the Bayesian Regularized Neural Network need to be slightly modified to take into account that the residual variance for $e_{i}$ in equation (5) is set to 1. The Appendix shows the computational details for estimating posterior modes for these parameters. One interesting output from this algorithm is the effective number of parameters η which can give some guidance about the number of neurons to include in the neural network (see, for example, Pérez‐Rodríguez et al., 2013). This parameter is computed as follows (see Appendix 1 for more details):

$$\eta \;=\;p-2\alpha Trace\left(H^{-1}\right),$$

where *p* is the number of elements in ***θ***,$\;\alpha \;=\frac{1}{2\sigma_{\theta }^{2}}\;$, $H\;=\frac{\partial^{2}}{\partial \theta \partial \theta^{t}}\;F\left(\theta \right)$, $F\left(\theta \right)=\frac{1}{2\sigma_{e}^{2}}\;\sum_{i\;=\;1}^{n}{\left(z_{i}-{\hat{z}}_{i}\right)}^{2}+\frac{1}{2\sigma_{\theta }^{2}}\sum_{l\;=\;1}^{p}\theta_{l}^{2}$. When this parameter shows little change for *S* and *S+*1 neurons, the more parsimonious model is preferred.

The GEM algorithm (Kärkkäinen & Sillanpää, 2013; Neal & Hinton, 1998) is similar to the GMAP algorithm. The ‘tractable’ parameters (latent variables and thresholds) are updated according to the conditional expectations for distributions given in (9) and (10) and the ‘intractable’ parameters are estimated using MAP, so the updating of ‘tractable’ and ‘untractable’ parameters is repeated until convergence. As the number of parameters to estimate increases, the GMAP algorithm becomes slow, and therefore, our preferred algorithm for fitting the proposed model is GEM. Appendix 2 shows computational details for implementing the GEM algorithm.

### 2.4 The software

The ordered probit model described above can be fitted using the BGLR library functions in R (Pérez & de los Campos, 2014). The ordered logit model proposed by Montesinos‐López et al. (2015b) can also be fitted in R (R Core Team, 2019); the authors provided the code in the supplementary materials included in the published paper. The GEM algorithm for fitting the ordinal Bayesian Regularized Neural Network proposed in this study was implemented using the C programming language (Kernighan & Ritchie, 1988) and the R statistical package (R Core Team, 2019) to speed up computations and facilitate user interaction with the software. We included the resulting routines for fitting the proposed model in the brnn package version 0.8 with the GEM algorithm (Pérez‐Rodríguez & Gianola, 2020), while the code for fitting the model with the GMAP algorithm is included in supplementary materials. We decided not to include it in the brnn package, as it is very slow.

### 2.5 Measurements of genome‐based prediction accuracy of models for ordinal data

In order to evaluate the proposed model's predictive ability, we proposed partitioning the data at random into the training and testing sets. The basic idea was to fit the model with the training data and then predict phenotypes (observed ordinal response) for the individuals in the testing set. In the case of continuous response, the model's predictive ability was measured by Pearson's correlation coefficient between observed and predicted phenotypical values or mean squared error prediction (MSEP).

However, in the case of categorical data, several metrics are widely used, such as sensitivity, specificity, ROC curve, etc. (Tharwat, 2018). Montesinos‐López et al. (2015b) suggested using the Brier score (BS) (Brier, 1950) because these authors argued that this statistic uses all the information contained in the predictive distribution. The Brier score can be computed as follows:

$$\mathrm{BS}\;=\frac{1}{2n}\;\sum_{i\;=\;1}^{n}\sum_{j\;=\;1}^{K}{\left({\hat{\pi }}_{ij}-d_{ij}\right)}^{2}$$

where *n* is the total number of observations in the set of interest, ${\hat{\pi }}_{ij}$ is the estimated probability that observation i=1,…,n belongs to class j=1,…,K, which can be computed using the adjusted model with the equations (6)‐(7) and $d_{ij}=\;1$, if observation *i* belongs to class *j* and $d_{ij}=\;0$ otherwise. As defined above, BS takes values between 0 and 1, and small values are associated with better prediction ability.

Other genome‐based prediction performance measures that are used with ordinal data are, for example, the misclassification error rate (MER), which is obtained by counting the number of classification errors and then dividing the result by the number of test cases. Mathieson (1995) argued that for ordinal responses, performance measures that take into account the difference between class numbers are preferred over MER due to the lack of better information. In this case, two performance measures that take into account this criterion are Mean Absolute Error (MAE) computed as $\frac{1}{n}\sum_{i\;=\;1}^{n}\left|y_{i}-{\hat{y}}_{i}\right|$ and Root Mean Square Error (RMSE), which can be obtained as $\sqrt{\frac{1}{n}\sum_{i\;=\;1}^{n}{\left(y_{i}-{\hat{y}}_{i}\right)}^{2}}$ (see da Costa & Cardoso, 2005, for more details). Pearson's correlation coefficient, widely used with continuous data, can be replaced by Sperman's rank correlation coefficient or Kendall's tau coefficient; here, we used Sperman's coefficient. It is important to note that the predicted class ${\hat{y}}_{i}$ in the testing set is obtained by first predicting the value of the latent variable ${\hat{z}}_{i}$ using the estimated parameters and associated covariates, and then ${\hat{y}}_{i}=\;j$ if ${\hat{\lambda }}_{j-1}<{\hat{z}}_{i}<{\hat{\lambda }}_{j}$, where $\hat{\lambda }$ is the vector of estimated threshold parameters.

### 2.6 Applying models to real data

#### 2.6.1 The Septoria dataset

Septoria is a fungus that causes leaf spot diseases in field crops, forage crops and vegetables. The Septoria dataset includes information for 268 wheat lines from CIMMYT (http://www.cimmyt.org) and was previously analyzed by Montesinos‐López et al. (2015b). The lines were planted in 2010 in Toluca, Mexico. The dataset includes information about disease severity, which was measured on an ordinal scale with four points. The lines were genotyped using Genotype by Sequencing (GBS; Poland et al., 2012). Markers were filtered by removing markers with more than 50% missing values, imputed using observed allelic frequencies, and further removing markers with minor allele frequency smaller than 0.05. After that, 6787 markers were still available for further analysis. The dataset can be downloaded from http://hdl.handle.net/11529/10254.

#### 2.6.2 The GLS dataset

Gray Leaf Spot (GLS) is a disease caused by the fungus *Cercospora zeae‐maydis*. This dataset consists of genotypic and phenotypic information for 278 maize lines from the Drought Tolerance Maize (DTMA) project of CIMMYT's Global Maize Program. The dataset was originally analyzed by Crossa et al. (2011), and re‐analyzed later by González‐Camacho et al. (2012), Montesinos‐López et al. (2015b) and Pérez‐Rodríguez et al. (2018) using different statistical models. The dataset includes information on disease severity measured on an ordinal scale with 5 points: 1 = no disease, 2 = low infection, 3 = moderate infection, 4 = high infection and 5 = totally infected. The lines were initially genotyped with 1,152 SNPs and re‐genotyped later with 55k SNPs using the Illumina platform. After removing SNPs with more than 10% missing values and imputing filtering markers with minor allele frequency smaller than 0.05, a total of 46,347 markers were still available for further analysis. The dataset can be downloaded from http://hdl.handle.net/11529/10254.

We evaluated the predictive ability of the proposed model by using 100 partitions generated at random with 90% of observations in the training set and the remaining 10% in the testing set. For each partition, we fitted the Bayesian Regularized Neural Network (BRNN) for ordinal data with two neurons. In order to expedite computations, we first computed a genomic relationship matrix (**G**) between centered and standardized genotypes (Lopez‐Cruz et al., 2015); we then performed the eigen‐value decomposition of **G**, that is, $G\;=\;{\Gamma \Lambda \Gamma }^{\prime }$, and used $\Gamma \Lambda^{\frac{1}{2}}$ (principal components) as our matrix of covariates to fit the models (see Gianola et al., 2011, for other computing strategies). The model was fitted using the GMAP and GEM algorithms. In the case of GMAP, inferences were based on 5000 MCMC iterations obtained after discarding 5000 samples that were taken as burn‐in, due to high computational times.

In the case of ordered probit regression, we used the same computational strategy as before for speeding up computations. We fitted the model using the BGLR package in R (Pérez & de los Campos, 2014), and inferences were based on 5000 MCMC iterations obtained after discarding 25,000 samples that were taken as burn‐in. For each partition, we computed several statistics that allowed us to assess the performance of the models; in this case, Brier score, MAE, RMSE, Spearman's correlation (r) and MER.

## 3 RESULTS

Table 1 shows the results for Septoria and GLS in Colombia, Harare and Mexico. Results in Table 1 show the five criteria for determining the best models for all of them (except the correlations), i.e., the lower the better, whereas for the correlations, the higher the better. Results show that the BRNNO model performed better than the probit model (BOPM), except in the case of BS criterion for the Septoria disease (BS = 0.3205). For all the other cases, the performance measured by criteria MAE, RMSE and MER was always smaller for BRNNO than for the ordered probit model (BOPM). In addition, it should be noted that the associated Spearman's rank correlation coefficient is higher for BRNNO than for the probit model (BOPM). The best prediction of the BRNNO algorithm occurred for disease GLS measured in Colombia using the GEM algorithm with 0.5745, 0.8683, 0.7295, and 0.4942 values for criteria MAE, RMSE, r, and MER, respectively. Furthermore, usually the second best model was also BRNNO (using either one of the two algorithms GEM or GMAP).

**TABLE 1:** Performance measures (BS, MAE, RMSE, r and MER) for the Bayesian Ordered Probit Model (BOPM) and Bayesian Regularized Neural Network for Ordinal data (BRNNO) for Septoria and GLS at three locations (Colombia, Harare and Mexico). Standard deviation (sd). GMAP denotes the Gibbs Maximum a Posteriori Algorithm and GEM is the Generalized EM algorithm

Model	Dataset/Location	Statistic	BS	MAE	RMSE	r	MER
BOPM	Septoria	Mean	0.3205	0.6138	0.8926	0.2742	0.5173
		Sd	0.0287	0.1429	0.1507	0.1650	0.1065
	GLS Colombia	Mean	0.3550	0.6741	0.9301	0.6963	0.5804
		Sd	0.0083	0.0719	0.0770	0.0629	0.0503
	GLS Harare	Mean	0.3458	0.6772	0.9342	0.4335	0.5792
		Sd	0.0070	0.0495	0.0440	0.0566	0.0398
	GLS Mexico	Mean	0.3359	0.6998	0.9308	0.4141	0.6142
		Sd	0.0167	0.0851	0.0822	0.0887	0.0640
BRNNO GMAP	Septoria	Mean	0.3562	0.6197	0.8961	0.3078	0.5256
		Sd	0.0518	0.1173	0.1376	0.1703	0.0839
	GLS Colombia	Mean	0.3299	0.5878	0.8734	0.7241	0.5061
		Sd	0.0291	0.0800	0.0967	0.0621	0.0549
	GLS Harare	Mean	0.3340	0.6510	0.9311	0.4971	0.5464
		Sd	0.0131	0.0536	0.0587	0.0694	0.0393
	GLS Mexico	Mean	0.3418	0.6063	0.9087	0.5541	0.5000
		Sd	0.0424	0.1024	0.1163	0.1160	0.0667
BRNNO GEM	Septoria	Mean	0.3355	0.5927	0.8742	0.3176	0.5023
		Sd	0.0513	0.1363	0.1474	0.2059	0.0991
	GLS Colombia	Mean	0.3279	0.5745	0.8683	0.7295	0.4942
		Sd	0.0162	0.0768	0.0935	0.0644	0.0564
	GLS Harare	Mean	0.3430	0.6574	0.9355	0.4916	0.5533
		Sd	0.0071	0.0508	0.0495	0.0616	0.0387
	GLS Mexico	Mean	0.3431	0.5910	0.8704	0.5503	0.5088
		Sd	0.0105	0.0978	0.1014	0.1021	0.0781

BS = Brier Score (the smaller the better); MAE = Mean Absolute Error (the smaller, the better); RMSE = Root Mean Square Error (the smaller, the better); r = Spearman correlation coefficient (the higher, the better); MER = Misclassification Error Rate (the smaller, the better).

Table 2 shows the number of parameters that should be estimated for the neural network and the estimated effective number of parameters. We fitted the models with *S = 3* neurons and concluded that it was not necessary to include more neurons.

**TABLE 2:** Number of parameters to estimate and the estimated effective number of parameters and corresponding standard deviation

Dataset	Number of parameters to estimate (biases, weights and connection strengths)	Effective number of parameters GMAP algorithm	Effective number of parameters GEM algorithm
Septoria	538	211.28 (4.33)	198.19 (2.27)
GLS Colombia	558	259.23 (1.90)	231.00 (4.63)
GLS Harare	516	237.31 (1.44)	221.63 (2.64)
GLS Mexico	524	230.48 (3.58)	159.76 (5.80)

**FIGURE 2:**
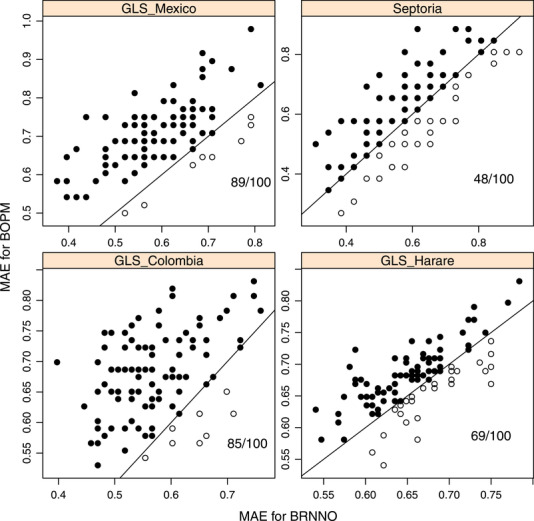
Scatter plot matrix of MAE for BRNNO and BOPM. Points above the 45° degree line are associated with the worst performance of the model on the y‐axis (BOPM) in comparison with the model presented on the x‐axis (BRNNO). When the worst model is BOPM, this is represented by a filled circle; however, when the worst model is BRNNO, it is represented by an open circle. The plots also show the number of times that BOPM is inferior to BRNNO as %

**FIGURE 3:**
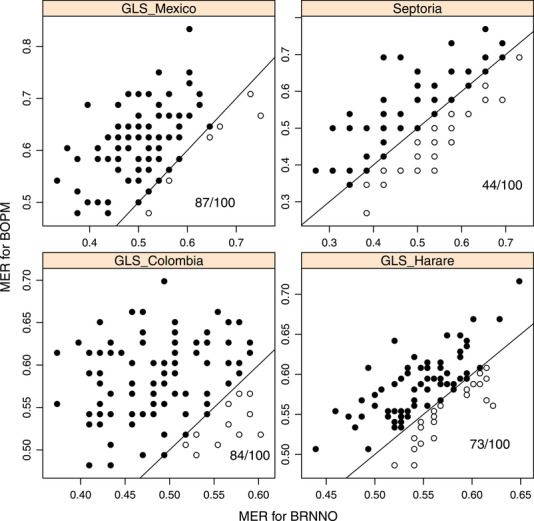
Scatter plot matrix of MER for BRNNO and BOPM. Points above the 45° degree line are associated with the worst performance of the model on the y‐axis (BOPM) in comparison with the model presented on the x‐axis (BRNNO). When the worst model is BOPM, it is represented by a filled circle, and when the worst model is BRNNO, it is represented by an open circle. The plots also show the number of times that BOPM is inferior to BRNNO as %

Figures 2 and 3 show the evaluation of metrics MAE and MER for Septoria and GLS in Colombia, Harare and Mexico, respectively, for each of the 100 partitions generated at random and obtained with the GEM algorithm. Results show similar patterns for the GMAP algorithm. These figures are a graphical representation of the summary shown in Table 1. The 45**°** line makes it possible to quickly compare the performance of the two models, from where it is clear that MAE and MER are worse for the probit model than for the proposed (BRNNO) model because they exhibit higher values.

For the MAE criterion, BRNNO was superior to BOPM in GLS because the latter exhibited higher MAE in 89% of times in Mexico, 85% of times in Colombia and 69% of times in Harare. The MAE for Septoria was slightly better for BOPM since BRNNO was worse in 52% of times. Similar patterns were observed in the case of MER, where BOPM was worse than BRNNO for GLS, MER was higher 87% of the times for Mexico, 84% of times in Colombia and 73% of times in Harare. Finally, in the case of Septoria, the BOPM performed slightly better than the proposed model since it had smaller MER values 56% of times.

## 4 DISCUSSION

Results suggest that the proposed model BRNNO performs better than the BOPM model for both algorithms, GMAP and GEM; however, GEM is several orders of magnitude faster than GMAP. For example, for the GLS dataset, the process took ∼8 minutes to fit the proposed model with the GEM algorithm in a computer with an intel quad core i7 processor at 2.8 GHz, whereas for 10,000 MCMC iterations with GMAP on the same computer, the process lasted ∼3 hours. Thus it is clear that computing times are reduced substantially by using the GEM algorithm.

As already mentioned, the single hidden layer neural network (BRNNO) proposed in this study generalizes the Bayesian ordered probit model (BOPM), as well as the Bayesian ordered logit model (BOLM) of Montesinos‐López et al. (2015b). It should be noted that the Brier score results from Montesinos‐López et al. (2015b) are not directly comparable with those obtained for the proposed BRNNO method in this study. However, the pooled BS of the real GLS data in Table 4 of Montesinos‐López et al. (2015b) showed a slightly higher BS of around 0.37 for BOLM and BOPM than the BS estimated for the BRNNO ranging from 0.33–0.35 for the three locations in Colombia, Mexico, and Harare (Table 1). The BS values for the Bayesian ordered probit model (BOPM) of this study for the three locations, Colombia, Harare, and Mexico, were 0.3550, 0.3458, and 0.3359, respectively.

Not many studies have been reported on the prediction of ordinal traits in genomic‐enabled prediction. Recently, Montesinos‐López et al. (2019) proposed a deep learning method for the simultaneous prediction of mixed phenotypes (binary, ordinal and continuous) in plant breeding. The proposed neural network BRNNO and the open‐source R package brnn referred in this study for the genomic‐enabled prediction of ordinal data is important due to the fact that there is a lack of genomic‐enabled studies predicting categorical data in plant breeding and simultaneously offering ready‐to‐use R software.

## 5 CONCLUSIONS

We introduced a neural network that generalizes existing models for the prediction of ordinal responses. We explored two algorithms (GEM and GMAP) that can be used to fit the proposed model. The GMAP algorithm is able to fit the model, but it is very slow because at each iteration, it is necessary to fit a Bayesian Regularized Neural Network using the latent variable as a response; additionally, it is necessary to have several thousands of MCMC iterations to make inferences. In contrast, although the GEM algorithm is also slow in the empirical evaluation, it requires much fewer iterations to converge; these results are in agreement with Kärkkäinen and Sillanpää (2013). Thus, we conclude there is ample room for improvement in both algorithms.

Regarding the genomic‐enabled predictive accuracy of the models, most of selected indexes (MAE, RMSE, r, MER), except the Brier score, favored the proposed BRNNO model, fitted either with the GMAP or the GEM algorithm. Improvements are limited, but consistent with the findings of other studies (Montesinos‐López et al., 2015b). We should point out that this approach could be applied not only in the GS context, but also in the context of conventional phenotype plant breeding for disease resistance and many other ordinal traits.

## Supporting information

Supplementary Material

Supplementary Material

Supplementary Material

## APPENDIX 1 GMAP ALGORITHM

### 1.1

Here we describe the algorithm for fitting the proposed Bayesian Regularized Neural Network for Ordinal Data (BRNNO). The proposed algorithm is known in statistical literature as GMAP (Kim et al., 2009); it combines the well‐known Gibbs Sampler algorithm (G) to sample from some random variables and an optimization algorithm to obtain the posterior mode (Maximum A Posteriori = MAP) of other parameters of the model. The algorithm can be summarized as follows:

1. Initialization step.
  $$\begin{array}{ccc} \mathrm{Initialize}\;\theta \; & = & \left(w_{1},\;\ldots ,w_{S};b_{1},\ldots ,b_{S};\beta^{\left[1\right]},\ldots ,\beta^{\left[S\right]}\right),\\ & & \;\sigma_{\theta }^{2},\lambda \;=\left\{\lambda_{1},\ldots ,\lambda_{K}\right\}\;,\\ & & \;Z\;=\;\left\{Z_{1},\ldots ,Z_{n}\right\}. \end{array}$$

G‐Step

1. Sample $Z_{i}|\;Y_{i}=\;j,\mathrm{else},\;i\;=\;1,\ldots ,n$; see equation (9).
2. Sample $\lambda_{j}|\mathrm{else}$, j=2,…,K; see equation (10).

MAP‐Step

1. Fit for the regularized neural network, using as a response variable the pseudo‐data generated in step 2 (see equation 5):
  $$\;Z_{i}=\sum_{m=1}^{s}w_{m}\;g\left(b_{m}+x_{i}^{t}\beta^{\left[m\right]}\right)+e_{i},$$

with $g\;\left(u\right)=\frac{2}{1+\exp(-2u)}\;-1$ the tangent hyperbolic activation function.

Steps 2–4 are repeated *B* times until convergence.

The steps necessary to fit the regularized neural network with pseudo‐data are essentially the same as those used to fit a Bayesian Regularized Neural Network for a continuous response; see, for example, Foresee and Hagan (1997), Okut et al. (2011), Gianola et al. (2011), Pérez‐Rodríguez et al. (2013). Here we summarize the basic algorithm:

1. Obtain the conditional posterior mode of the elements in ***θ***, setting $\sigma_{e}^{2}=\;1$ and assuming that $\sigma_{\theta }^{2}$ is known. These modes are obtained by minimizing the augmented sum of squares:
  $$F\;\left(\theta \right)=\frac{1}{2\sigma_{e}^{2}}\;\sum_{i\;=\;1}^{n}e_{i}^{2}+\frac{1}{2\sigma_{\theta }^{2}}\sum_{l\;=\;1}^{p}\theta_{l}^{2},$$

where $e_{i}=z_{i}\;-{\hat{z}}_{i}$ is the prediction error and *p* is the number of elements in vector ***θ***. The augmented sum of squares can be minimized using the Levenberg‐Marquardt algorithm (Levenberg, 1944; Marquardt, 1963).

1. Update the variance component $\sigma_{\theta }^{2}$ by maximizing $p(z|\sigma_{\theta }^{2})$. Because of non‐linearity, the marginal log‐likelihood, $\log p(z|\sigma_{\theta }^{2}),$ is not expressible in closed form, but can be approximated as:

with $\Sigma \;=\frac{\partial^{2}}{\partial \theta \partial \theta^{t}}\;F\left(\theta \right)$, $\alpha \;=\frac{1}{2\sigma_{\theta }^{2}}\;$. The α parameter that maximizes the marginal likelihood is updated iteratively, $\alpha_{new}=\frac{\eta }{\sum_{l=1}^{p}\theta_{l}^{2}}\;$ and $\eta \;=\;p-2\alpha_{old}Trace\left(H^{-1}\right)$, with $\alpha_{new}$ the updated value of the parameter and $\alpha_{old}$ the value of the parameter in the previous iteration.

The algorithm described above was implemented in R and the C programming language (Kernighan & Ritchie, 1988), and is included as electronic supplementary material, but was not included in the brnn package because it is very slow. The user can fit a Bayesian Regularized Neural Network by providing data to the R function **brnn_ordinal_mcmc**, which internally combines interpreted and compiled codes to fit the model. Upon successful execution, the routine returns an R object with MCMC samples for {Z,λ} and the posterior mode for $\left\{\theta ,\sigma_{\theta }^{2}\right\}$ obtained by fitting the Bayesian regularized neural network with ***Z*** and ***λ*** fixed to the posterior mean of those parameters obtained from MCMC samples. MCMC samples can be obtained for posterior distributions for thresholds, i.e., $p(\lambda_{j}|\mathrm{data}).$ The output object also allows the prediction of new observations (e.g., in a testing set for cross‐validation) using estimated network parameters.

## APPENDIX 2 GEM ALGORITHM

### 2.1

The GEM algorithm (Neal & Hinton, 1998; Kärkkäinen & Sillanpää, 2013) is similar to the GMAP algorithm. The ‘tractable’ parameters (latent variables and thresholds) are updated according to the conditional expectations for distributions given in (9) and (10) and the ‘intractable’ parameters are estimated using MAP, so updating of ‘tractable’ and ‘untractable’ parameters is repeated until convergence.

The algorithm can be summarized as follows:

1. Initialization step.
  $$\begin{array}{ccc} \mathrm{Initialize}\;\theta \; & = & \left(w_{1},\;\ldots ,w_{S};b_{1},\ldots ,b_{S};\beta^{\left[1\right]},\ldots ,\beta^{\left[S\right]}\right)\;,\\ & & \;\sigma_{\theta }^{2},\lambda \;=\left\{\lambda_{1},\ldots ,\lambda_{K}\right\}\;,\\ & & \;Z\;=\;\left\{Z_{1},\ldots ,Z_{n}\right\}. \end{array}$$

E‐Step

1. Update the latent variables $Z_{i}|\;Y_{i}=\;j,\mathrm{else},\;i\;=\;1,\ldots ,n$, by replacing the current values with the expected values from truncated normal distribution given in (9).
2. Update the thresholds Sample $\lambda_{j}|\mathrm{else}$, j=2,…,K, by replacing the current values with the expected values from the uniform distribution given in (10).

MAP‐Step

1. Fit the regularized neural network using the pseudo‐data generated in step 2 as a response variable (see equation 5):
  $$\;Z_{i}=\sum_{m=1}^{s}w_{m}\;g\left(b_{m}+x_{i}^{t}\beta^{\left[m\right]}\right)+e_{i},$$

with $g\;\left(u\right)=\frac{2}{1\;+\;\exp(-2u)}\;-1$ the tangent hyperbolic activation function.

Steps 2–4 are repeated until convergence.
